# 3D flower-like molybdenum disulfide modified graphite felt as a positive material for vanadium redox flow batteries†

**DOI:** 10.1039/d0ra02541k

**Published:** 2020-05-04

**Authors:** Lei Wang, Shuangyu Li, Dan Li, Qinhao Xiao, Wenheng Jing

**Affiliations:** State Key Laboratory of Materials-Oriented Chemical Engineering, College of Chemical Engineering, Nanjing Tech University Nanjing 211816 China jingwh@njtech.edu.cn; Jiangsu Jiayi Thermal Power Co., Ltd. Changzhou 213200 China

## Abstract

3D flower-like molybdenum disulfide microsphere modified graphite felt (MoS_2_/GF) with excellent electrocatalytic activity and redox reversibility for the VO^2+^/VO_2_^+^ couple is successfully fabricated by a facile hydrothermal method. The results show that the hydrothermal reaction time has a deep influence on the MoS_2_ structure; an open 3D flower-like MoS_2_ structure with a layer spacing of 0.63 nm is uniformly grafted on the GF surface for a reaction time of 36 h. With the presence of MoS_2_, the total resistance (1.58 Ω) and charge transfer resistance (0.01 Ω) of MoS_2_/GF-36 are smaller than that of the heat treated GF (2.04 Ω and 11.27 Ω, respectively), indicating that the electrode has better conductivity and more favorable electron transfer ability. As expected, a significant increase in the capacity and energy efficiency is obtained with the MoS_2_/GF-36 electrode. These satisfactory results are attributed to the 3D flower-like structure on the surface of the electrode, which increases the contact area between the electrode and the electrolyte. More importantly, the MoS_2_/GF electrode with excellent stability has great application prospect in vanadium redox flow batteries (VRFBs).

## Abstract 2

The open flower-like structure facilitates vanadium ion transport. The capacity and efficiency of a battery using MoS_2_/GF are dramatically increased.

## Graphical abstract

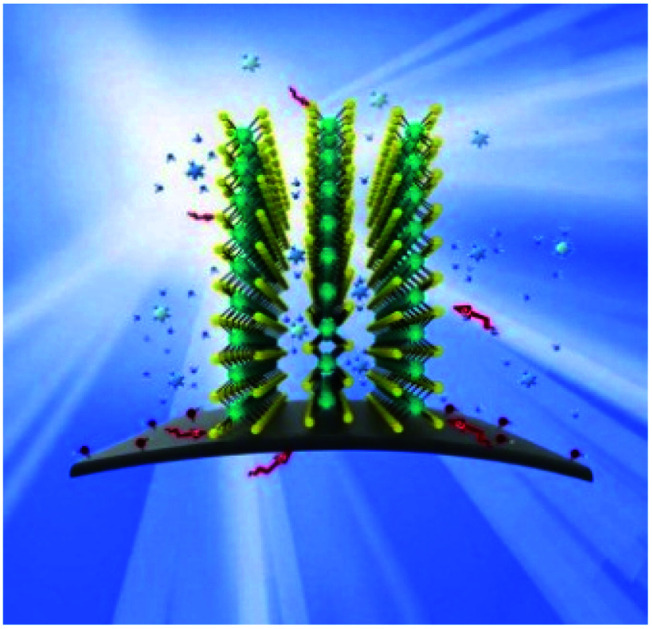

## 1. Introduction

As global environmental pollution and energy shortages caused by the use of fossil fuels become increasingly prominent, the global strategic deployment of clean energy, such as solar energy and wind energy, has been accelerating.^1–3^ However, due to the unstable and intermittent nature of clean energy, the deployment of large-scale energy storage systems (ESSs) is in high demand. Among different ESSs, redox flow batteries (RFBs) are considered to be one of the most promising technologies because of their attractive features, including their high energy efficiency, long cycle life, safety and stability.^4–6^ The researchers found many types of batteries, such as Ce–V, Cr–Fe, Ce–Ce, V–V, *etc.*^7–9^ Among them, the all-vanadium redox flow battery (VRFB) has received considerable attention and is at the commercial demonstration stage, which employs the same elements in two semi-batteries, decreasing the risk of the cross-contamination of the electrolyte.^10^

A VRFB mainly consists of three components, including the electrolyte, the separation membrane and the electrode. The redox reaction performs on the surface of the electrode; thus a high activity electrode is desirable for improving the power density and energy density of a VRFB. It has demonstrated that the redox reaction (V^2+^/V^3+^) at the negative electrode is reversible and present fast reaction kinetics due to having only one electron transfer. By contrast, the positive reaction is more complex (one electron and two protons are transferred) and presents relatively slow reaction kinetics, so the reaction at the positive electrode (VO^2+^/VO_2_^+^) is the rate determination step.^11–14^ In addition, the range of the electrocatalytic materials is limited by the acidic medium and the high redox potential (1.0 V) of the VO^2+^/VO_2_^+^ couple.^15,16^ Therefore, it is critically important to develop a high activity and stable electrode, especially the positive electrode, to enhance the battery's performance.

Currently, polyacrylonitrile (PAN) graphite felt (GF) is widely used as a VRFB electrode due to its good stability in strong acid solutions, wide operating potential range, high conductivity and high surface area.^17,18^ In spite of these merits, GFs commonly require further functionalization treatments for fast reaction kinetics and better wettability. As a result, various surface treatments have been made to improve the electrochemical activity of the electrode by increasing the oxygen functional groups or nitrogen functional groups, such as thermal treatment, acid treatment, ammonia treatment or depositing catalysts onto the surface of electrode.^19–28^ Among those, intensive efforts have been focused on depositing catalysts onto the surface of the electrode due to the flexibility in adjusting the catalytic activity and surface morphology. Recently, MoS_2_ nanosheets, which have a typical graphite-like layered structure, have received significant attention and are considered to be a promising anode material due to their high electrocatalytic activity and large interlayer spacing (0.62 nm). It has been demonstrated that grafting MoS_2_ onto carbon fiber can enhance the electron transfer and increase their available and accessible active surface area.^29,30^ Nevertheless, the low inherent electronic conductivity of MoS_2_ would cause serious polarization and a low electrode utilization efficiency. In this regard, tailoring and optimizing the nanostructure of MoS_2_ are necessary in order to achieve a high electrochemical performance. For instance, flower-like MoS_2_/C nanospheres in an Na-ion battery anode present a high capacity and long cycle life.^31^ Additionally, increasing the layer spacing would reduce the ion diffusion resistance. Therefore, to be applied in VFRBs, the nanostructure of MoS_2_ should be elaborately designed to obtain a high battery efficiency.

In this study, we successfully synthesized low-cost 3D flower-like MoS_2_ as an electrocatalyst on the surface of GF to enhance the electrochemical activity of the VO^2+^/VO_2_^+^ redox reaction *via* a facile hydrothermal method. It has been reported that the hydrothermal reaction time plays an important role in controlling the MoS_2_, so the influence of the reaction time on the structure and electrode performance was carefully investigated.^32,33^ For applications in the VRFB as an anode, the vanadium species reaction activity, the diffusion coefficient and the performance of a single VRFB cell were systematically studied to provide us with an insight into the effect of the MoS_2_ structure on the electrochemical performance.

## 2. Experimental procedures

### 2.1 Electrode preparation

All commercially available chemicals for this experiment were analytical grade and used directly without further purification. To avoid impurities and enhance wettability, commercial GFs (Shanghai Hongsheng Industrial Co., Ltd.) were sonicated in ethanol for 30 min, washed with deionized water and dried at 80 °C. Finally, the GFs were calcined in a muffle furnace at 400 °C for 2 h and marked as H-GFs.

For the preparation of the molybdenum disulfide doped graphite felt (MoS_2_/GF) electrodes, 0.48 g of Na_2_MoO_4_·2H_2_O and 0.6 g of thiourea (CH_4_N_2_S) were dissolved into 60 mL of deionized water under strong stirring. Then the H-GFs were infiltrated into the prepared solution and reacted at 200 °C for 24, 36 and 48 h in a 100 mL Teflon-line stainless-steel autoclave, denoted as MoS_2_/GF-*X* (*X* = 24, 36, 48). After cooling down, the electrodes were washed with a large amount of deionized water and ethanol, and dried at 80 °C.

### 2.2 Physicochemical characterization

The surface morphology and elemental mapping studies of the obtained electrodes were investigated *via* a scanning electron microscopy (SEM) and an energy dispersive X-ray spectroscopy (EDX) (Hitachi S-4800 microscope, Japan). A transmission electron microscope (TEM) and high-resolution transmission electron microscopy (HRTEM) images were obtained using a TF20, Jeol 2100F microscope. X-ray diffraction (XRD) measurements were performed on a Japanese science X-ray diffractometer (Miniflex 600X) with a Cu Kα radiation source (*λ* = 1.5418 Å), scanning between 5° and 80° (2*θ*) at a scan rate of 15° min^−1^. The Raman spectrum was recorded on a LabRAM HR Raman microscope. The X-ray photoelectron spectroscopy (XPS) analyses were performed with a ThermoFisher ESCALAB 250XiXPS system.

### 2.3 Electrochemical measurements

Cyclic voltammetry (CV) and electrochemical impedance spectroscopy (EIS) were performed on a GAMRY Reference 3000 electrochemical workstation using a three-electrode configuration in a 0.1 M VOSO_4_·3H_2_O and 3 M H_2_SO_4_ solution, which consisted of a MoS_2_/GF-*X* as the working electrode (1 cm × 1 cm), a platinum sheet as the counter electrode, and a saturated calomel electrode (SCE) as the reference electrode. The CV test was conducted from 0 to 1.6 V *vs.* the SCE at scan rates ranging from 1–30 mV s^−1^. The EIS test was conducted under an open circuit potential (OCP) with an excitation signal of 10 mV in the frequency range from 10^5^ to 10^−2^ Hz. For comparison, the H-GF was also studied and all the electrochemical characterizations were performed at room temperature in an N_2_ atmosphere.

### 2.4 VRFB single cell test

**Fig. 1:**
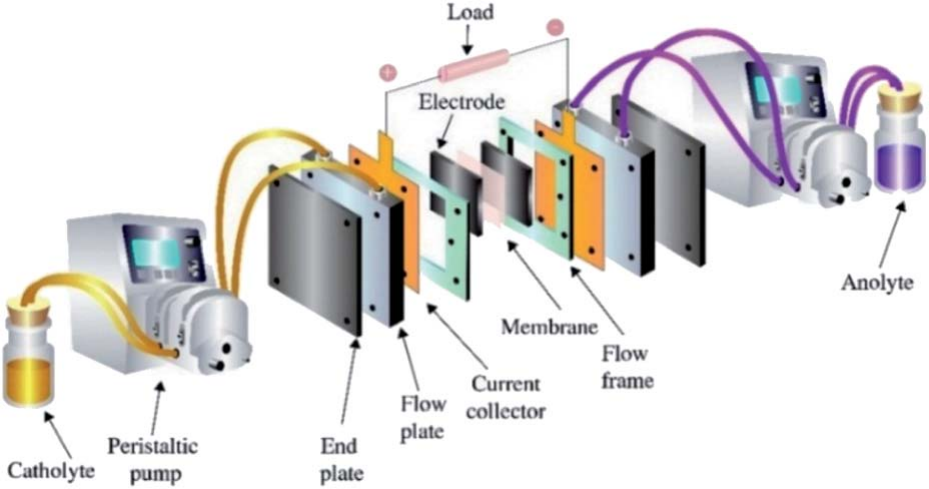
Schematic diagram of the vanadium redox flow battery.

The structure of the VRFB is shown in Fig. 1, and mainly consists of electrolytes, electrodes, various plates, ion exchange membrane, tanks and pumps. Among those, MoS_2_/GF-36 (3 cm × 3.5 cm) was employed as the positive electrode, while H-GF was used as the negative electrode. A Nafion 117 (real working area is 3 cm × 3.5 cm, DuPont, USA) membrane was utilized as the separator. The electrolyte consisted of 1.5 M VOSO_4_·3H_2_O and 3 M H_2_SO_4_ with a volume of 15 mL. The cell test was performed on a BTS-5V6A battery testing system (Shenzhen Newware Technology Electronics Co., Ltd.) with a potential range of 0.75–1.75 V. The charge–discharge test was conducted at current densities ranging from 30 to 150 mA cm^−2^. For comparison, a VRFB with H-GF as the positive electrode was also studied. Furthermore, all the VRFB cell tests were performed at room temperature in an N_2_ atmosphere.

## 3. Result and discussion

### 3.1 Physicochemical characterization

**Fig. 2:**
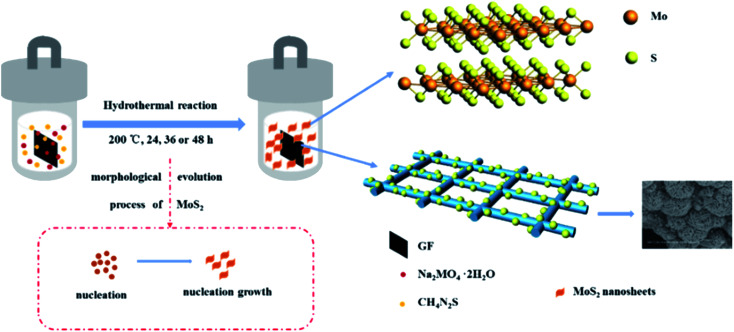
Schematic illustration of the synthesis of MoS_2_/GF-*X* electrodes.

3D flower-like molybdenum disulfide modified graphite felt (MoS_2_/GF) was directly fabricated *via* a simple one-step hydrothermal method, for which the schematic illustration is shown in Fig. 2. During the reaction process, thiourea is hydrolyzed to H_2_S, NH_3_ and CO_2_, and H_2_S is further hydrolyzed to H^+^ and S^2−^ under the high temperature and pressure environment. Then, Mo^6+^ is reduced by the presence of H^+^ and the precipitate particle of MoS_2_ is formed. The detailed reaction process is expressed by eqn (1)–(3). To further form the MoS_2_ microspheres, the particle mainly experiences a three-stage process, including rapid nucleation, orientation aggregation of nanosheets and the self-assembly of microsphere structures.^34^ In this case, the molar ratio of Na_2_MoO_4_·2H_2_O to CSN_2_H_4_ is 1 : 4, a large amount of MoS_2_ nuclei is formed in the first phase, and then the MoS_2_ nuclei develop to nanosheets because of the different growth rates of the crystal faces. Subsequently, to reduce their surface energy, the MoS_2_ nanosheets gather together and wrap around to form flower-like microspheres.

1 CS(NH_2_)_2_ + 2H_2_O → CO_2_ + 2NH_3_ + H_2_S

2 H_2_S → 2H^+^ + S^2−^

3 4MoO_4_^2−^ + 9S^2−^ + 24H^+^ → 4MoS_2_ + SO_4_^2−^ + 12H_2_O

**Fig. 3:**
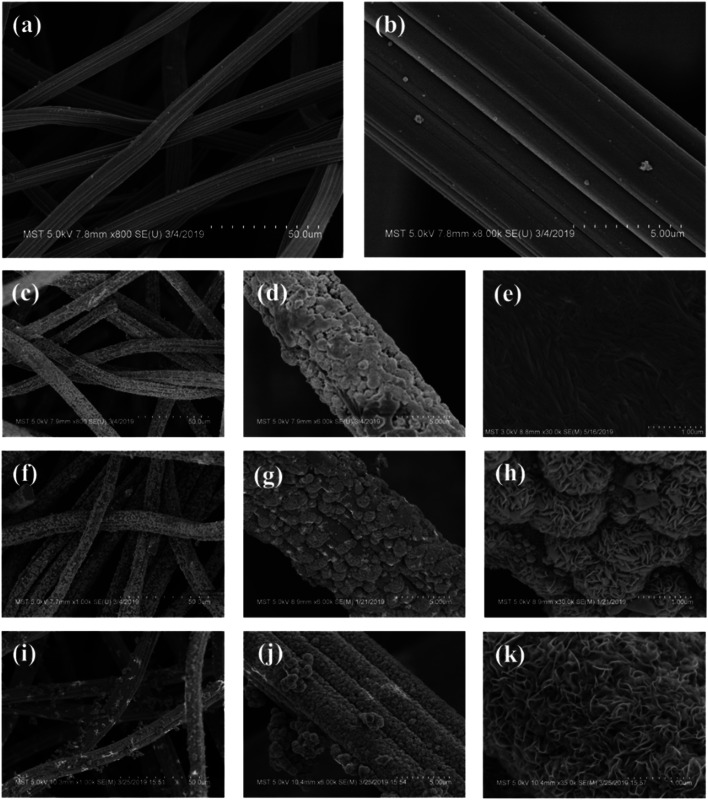
SEM images of (a and b) H-GF, (c–e) MoS_2_/GF-24, (f–h) MoS_2_/GF-36 and (i–k) MoS_2_/GF-48 electrodes.

To verify this, the surface morphology of the H-GF and MoS_2_/GF-*X* electrodes are detected with the results shown in Fig. 3. As observed, H-GF presents a typical interconnected network structure and its surface is defect-free and smooth. For the MoS_2_/GF-24 electrode, the surface of the GF is covered by a large number of MoS_2_ nanosheets. However, the nanosheets do not have an adequately developed flower structure. As the reaction time is extended to 36 h, uniform and dense MoS_2_ flower-like microspheres with diameters of ∼1 μm are observed on the GF surface, and the microsphere consists of tens of nanosheets from the high-magnification SEM image (Fig. 3(h)), which favors the adsorption and transfer of vanadium ions. However, the MoS_2_ nanosheets are non uniformly dispersed on the surface of the GF; most of the GF fibers cover many layers of MoS_2_ nanosheets and the 3D flower-like MoS_2_ spheres disappear when the reaction time is further extended to 48 h. The possible reason is that the accumulation of the H^+^ with the reaction time causes the charge shielding effect that a large amount of H^+^ gather on the edge of the nanosheet, resulting in MoO_4_^2−^ and S^2−^ only reacting with the outer layer of H^+^ to form MoS_2_. Therefore, the growth of the MoS_2_ nanosheets is inhibited to some extent, and finally leads to the disappearance of the petals. Based on this result, it is not difficult to determine that the optimum reaction time for obtaining 3D flower-like spheres on the surface of the GF is 36 h.

**Fig. 4:**
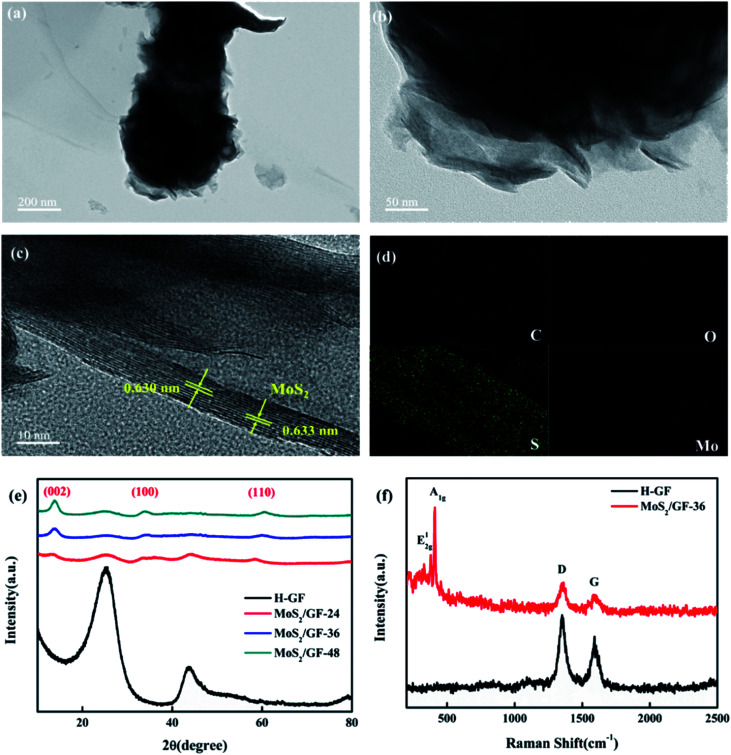
(a and b) TEM and (c) HRTEM images of the MoS_2_/GF-36 electrode. (d) EDX elemental mapping analysis of the MoS_2_/GF-36 electrode. (e) XRD patterns of the H-GF and MoS_2_/GF-*X* electrodes. (f) Raman spectra of the H-GF and MoS_2_/GF-36 electrodes.

Thereafter, the structure of MoS_2_/GF-36 is further analyzed *via* TEM and HRTEM, the results of which are shown in Fig. 4(a)–(c). It can be seen from the images that the MoS_2_ on the GF present a typical layered structure with an interlayer distance of 0.63 nm, corresponding to the (002) planes of MoS_2_.^35^ This layered structure can increase the available and accessible active surface area to electrolyte and enhance the redox reaction of the vanadium ions. Moreover, the MoS_2_/GF-36 consists of four elements, including Mo, S, O and C according to the EDX elemental spectra, wherein the four elements are evenly distributed on the GF surface (Fig. 4(d)).

The crystal structure of the H-GF and MoS_2_/GF-*X* electrodes are investigated by their XRD patterns (Fig. 4(e)). For the H-GF electrode, two peaks at 25.1° and 43.2° are observed, corresponding to the (002) and (100) planes of the GF, respectively.^36^ In the presence of the MoS_2_, new characteristic peaks at 13°, 33° and 59° appear, respectively corresponding to the (002), (100) and (110) crystal planes of MoS_2_, indicating the synthesized MoS_2_ presents a hexagonal crystal structure.^37^ Furthermore, the crystallinity of MoS_2_ is enhanced with an increase in the reaction time. It is worth noting that the diffraction peaks of the (100) and (110) crystal planes of the MoS_2_/GF-36 electrode are not obvious, and the diffraction peaks of the (002) crystal plane become obvious, indicating that the MoS_2_ nanosheets of the MoS_2_/GF-36 electrode are relatively thin and have more exposed edge structures. Further evidence for MoS_2_ grafting on the GF surface is provided by the Raman spectra (Fig. 4(f)). The GF present two different peaks at 1350 cm^−1^ and 1592 cm^−1^, corresponding to the D-band and the G-band of the GF, respectively.^38,39^ For the MoS_2_/GF-36 electrode, the in-plane E^1^_2g_ and the out-of-plane A_1g_ of MoS_2_ with typical hexagonal layered structures appear, whose characteristic peaks are at 376 cm^−1^ and 403 cm^−1^, respectively.^40^ This is consistent with the results of the XRD and SEM, and further confirms that the 3D flower-like MoS_2_ has successfully grown on the surface of the GF.

**Fig. 5:**
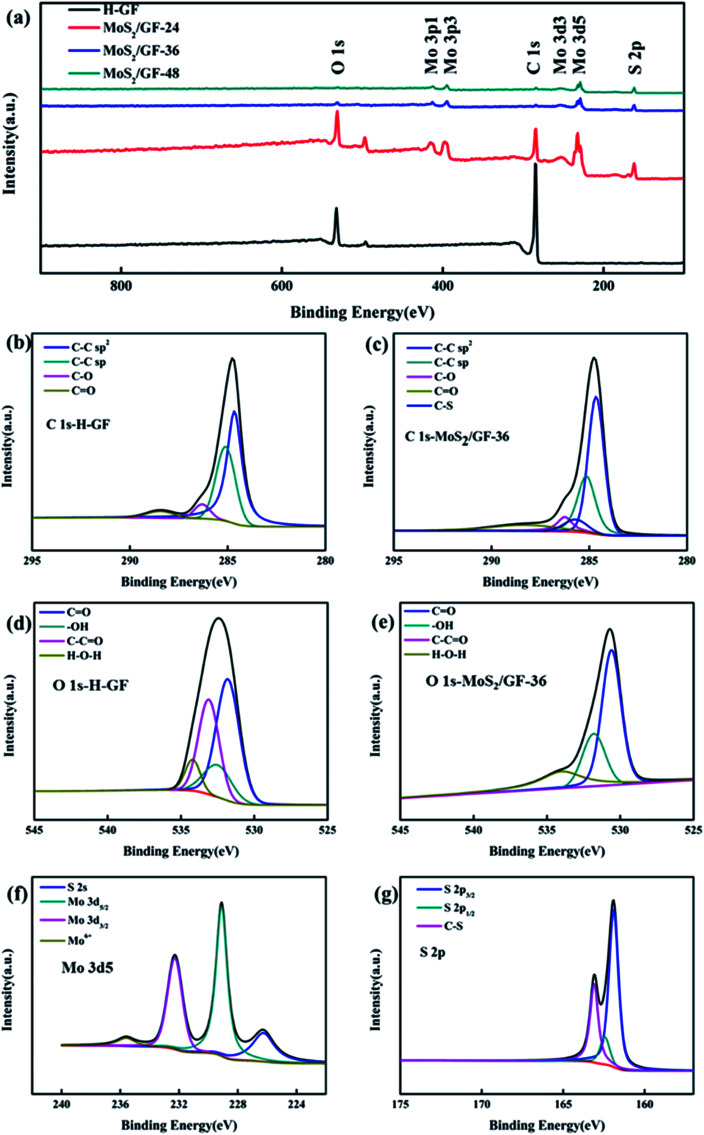
(a) The XPS of H-GF and MoS_2_/GF-*X* electrodes and curve-fitting narrow-scan XPS spectra for (b) C 1s of H-GF, (c) C 1s of MoS_2_/GF-36, (d) O 1s of H-GF, (e) O 1s of MoS_2_/GF-36, (f) Mo 3d5 of MoS_2_/GF-36, and (g) S 2p of MoS_2_/GF-36.

The local elemental compositions of the electrodes are analyzed *via* XPS (Fig. 5). As shown in Fig. 5(a), the GF consists of two elements, namely, C and O. For the MoS_2_/GF-*X* electrode, new peaks corresponding to the Mo and S elements are observed. For further insight into the binding mode of GF to MoS_2_ and the composition of oxygen-containing functional groups on GF, the spectra of C 1s and O 1s are analyzed, with the results shown in Fig. 5(b)–(e) and Table 1. According to the C 1s spectra of MoS_2_/GF-36 electrode, the C functional groups mainly consist of C–C sp^2^, C–C sp, C–O, C=O and a surplus peak that can be ascribed to C–S bond.^41–44^ This result fully demonstrates that MoS_2_ was successfully grafted onto GF. Furthermore, besides the C–S bond in MoS_2_/GF-36, GF and MoS_2_ may be firmly stacked by interlayer van der Waals attraction similar to graphene because of the surface energy minimization.^43,45,46^ Based on the O 1s spectrum, it can be convoluted into four peaks of C=O, –OH, C–C=O and H–O–H,^13,16,47^ and the fitting results show that the GF with grafted MoS_2_ flower-like microspheres significantly enhances the –OH content, which is attributed to the C–C=O bond cleavage and the synthesis of the –OH bond. It has been demonstrated that an increase in the oxygen-containing functional groups on the surface of the electrode can provide more active sites and enhance the VO^2+^/VO_2_^+^ redox reaction.^48–50^ Therefore, the prominent enhancement of –OH, which is a primarily important precursors for electron transfer in VO^2+^/VO_2_^+^ redox couples, can be beneficial for improving the performance of the vanadium battery.

**Table 1:** Contents (%) of various functional groups obtained from the curve fitting of the O 1s spectra

Electrode	C=O (%)	–OH (%)	C–C=O (%)	H–O–H (%)
H-GF	46.86	14.35	31.22	7.57
MoS_2_/GF-36	55.56	23.37	0	21.07

In addition, the Mo 3d spectrum is also analyzed, where the four different peaks respectively correspond to the S 2s of MoS_2_, the Mo 3d_5/2_ and Mo 3d_3/2_ of Mo^3+^ in MoS_2_ and the Mo 3d of MoO_3_, which may be attributed to the oxidation of the sample in air (Fig. 5(f)).^40^ Thereafter, the sulfur species are determined by the XPS S 2p spectrum, which has two peaks: the S 2p_3/2_ and S 2p_1/2_ lines of MoS_2_, indicating the presence of bridged S_2_^2−^ or apical S^2−^, respectively.^51–54^ It is noteworthy that the extra peaks in S 2p plot of MoS_2_/GF-36 can be assigned to C–S bond,^41,44,55^ which is in accord with the XPS results of C 1s. Combined with the above SEM, XRD, and TEM results, it is not difficult to determine that the 3D flower-like MoS_2_ microsphere modified GF electrode could absorb more vanadium ions and enhance the ion transfer and VO^2+^/VO_2_^+^ redox reaction.

### 3.2 Electrochemical characterization

**Fig. 6:**
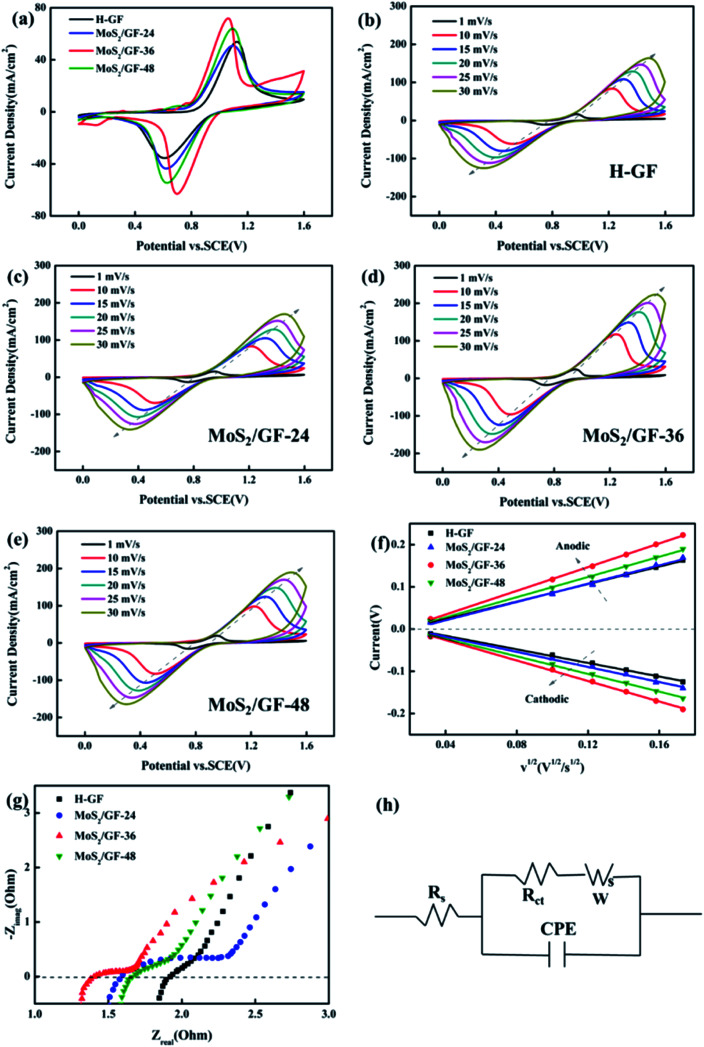
(a) Cyclic voltammograms obtained from an aqueous solution containing 0.1 M VOSO_4_ in an electrolyte of 3 M H_2_SO_4_ using the H-GF, MoS_2_/GF-24, MoS_2_/GF-36 and MoS_2_/GF-48 electrodes at a scan rate of 5 mV s^−1^. CV curves using (b) H-GF, (c) MoS_2_/GF-24, (d) MoS_2_/GF-36 and (e) MoS_2_/GF-48 in 0.1 M VOSO_4_ + 3 M H_2_SO_4_ solutions at different scan rates (1–30 mV s^−1^). (f) Plots of the redox peak current *vs.* the square root of the scan rate with different electrodes from 1 to 30 mV s^−1^. (g) EIS curves of H-GF and MoS_2_/GF-*X* electrodes in 0.1 M VOSO_4_ and 3 M H_2_SO_4_ solutions under an OCP. (h) Equivalent circuit diagram of the VRFB.

To further experimentally verify the enhancement of the VO^2+^/VO_2_^+^ redox reaction by the MoS_2_/GF-*X* electrodes, the CV of the electrodes is investigated and shown in Fig. 6(a). Several parameters are employed to evaluate its catalytic activity including the peak potential separation (Δ*E*_p_ = *E*_pa_ − *E*_pc_), redox onset potential, peak current density (*I*_pa_ and *I*_pc_) and peak current density ratio (*I*_pa_/*I*_pc_), with the detailed parameters summarized in Table 2. Significant differences between all the electrodes are observed. The MoS_2_/GF-*X* electrodes almost present high anodic/cathodic peak current, and small Δ*E*_p_ and *I*_pa_/*I*_pc_, indicating the improvement of the electrocatalytic activity and reaction reversibility, especially for the MoS_2_/GF-36 electrode. In addition, the MoS_2_/GF-36 electrode delivers the smallest onset potentials of the oxidation and the highest onset potentials of the reduction process, suggesting an enhancement of the electrocatalytic kinetic of the oxidation and reduction process. The optimal electrochemical activity and reversibility of the MoS_2_/GF-36 electrode verify that the 3D flower-like MoS_2_ microsphere has a positive effect for the VRFB battery.

**Table 2:** Electrochemical properties obtained from cyclic voltammetry of the VO^2+^/VO_2_^+^ reaction on various electrodes

Electrode	*I*_pa_ (mA cm^−2^)	*I*_pc_ (mA cm^−2^)	*E*_pa_ (V)	*E*_pc_ (V)	*I*_pa_/*I*_pc_	Δ*E*_p_ (V)
H-GF	53.79	−35.53	1.13	0.61	1.51	0.52
MoS_2_/GF-24	50.81	−43.61	1.10	0.62	1.17	0.48
MoS_2_/GF-36	71.85	−63.04	1.06	0.70	1.14	0.36
MoS_2_/GF-48	64.03	−54.57	1.09	0.63	1.17	0.46

To further investigate the effects of the MoS_2_ catalysts on the kinetics of the VO^2+^/VO_2_^+^ redox reaction, a series of CV plots for various electrodes at different scan rates from 1 to 30 mV s^−1^ are recorded and shown in Fig. 6(b)–(e). It can be seen from Fig. 6(f) that the anode and cathode peak currents of all the electrodes are linear with the square root of the scanning rate (*ν*^1/2^), thus the ion diffusion coefficient can be estimated according to eqn (4).^54,56,57^4
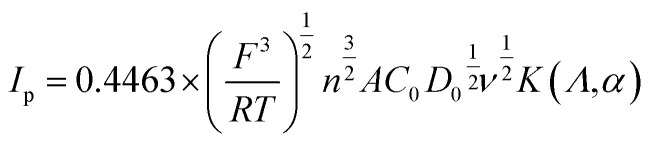
where *I*_p_, *F*, *R*, *T*, *A*, *C*_0_ and *D*_0_ are the peak current, Faraday constant, gas constant, Kelvin temperature, the surface area of the electrode, the concentration of the active material and the diffusion coefficient, respectively. *K*(*Λ*,*α*) is related to the degree of irreversibility. Since *I*_p_ is proportional to *ν*^1/2^, *K*(*Λ*,*α*) can be regarded as a constant within the allowable measurement range (*α* = 0.5, *K*(*Λ*,*α*) = 0.8). Therefore, eqn (4) can be simplified at 25 °C by considering the single electron transfer process of the VO^2+^/VO_2_^+^ quasi-reversible reaction as follows.^54,56,57^5
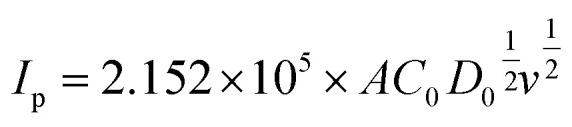


Based on the above equation, the diffusion coefficient of the electrodes can be calculated and summarized in Table 3. As observed, the *D*_0_ of each electrode for VO_2_^+^ → VO^2+^ is MoS_2_/GF-36 > MoS_2_/GF-48 > MoS_2_/GF-36 > H-GF, and a similar trend is found for VO^2+^ → VO_2_^+^. This illustrates that the MoS_2_/GF-36 electrode with a 3D flower-like structure enhances the ion transfer in the electrode and presents the best electrochemical activity for the VO^2+^/VO_2_^+^ redox reaction.

**Table 3:** The diffusion coefficient (*D*_0_) of the H-GF and MoS_2_/GF-*X* electrodes for the VO_2_^+^/VO^2+^ reaction

Electrode	*D*_0_ (10^−8^ cm^2^ s^−1^) for VO_2_^+^→ VO^2+^	*D*_0_ (10^−8^ cm^2^ s^−1^) for VO^2+^→ VO_2_^+^
H-GF	2.58	1.56
MoS_2_/GF-24	2.80	1.91
MoS_2_/GF-36	4.74	3.54
MoS_2_/GF-48	3.44	2.63

To understand the electronic charge transfer mechanism in the prepared electrodes, EIS measurements were performed at the open circuit voltage (OCP). All the obtained Nyquist plots (Fig. 6(g)) are composed of a semicircle portion in the high-frequency region and a linear portion in the low-frequency region, indicating that the redox process on the electrode surface is a mix that is controlled by the charge transfer and diffusion steps. Fig. 6(h) shows the equivalent circuit diagram of the Randles circuit and the fitting date are listed in Table 4. *R*_s_ represents the total resistance of the electrolyte and electrode, *R*_ct_ represents the charge transfer resistance, CPE is the constant-phase element, which represents the double-layer capacitance of the electrode/solution interface and W is the Warburg impedance. Among them, the MoS_2_/GF-36 electrode has the smallest *R*_s_ and *R*_ct_, suggesting it has good conductivity and fast electron transfer.

**Table 4:** Fitting data of the Nyquist plots obtained from the EIS results of Fig. 6(g)

Electrode	H-GF	MoS_2_/GF-24	MoS_2_/GF-36	MoS_2_/GF-48
*R*_s_ (Ω)	2.04	2.13	1.58	1.81
*R*_ct_ (Ω)	11.27	20.96	0.01	0.31

### 3.3 VRFB single cell performance

**Fig. 7:**
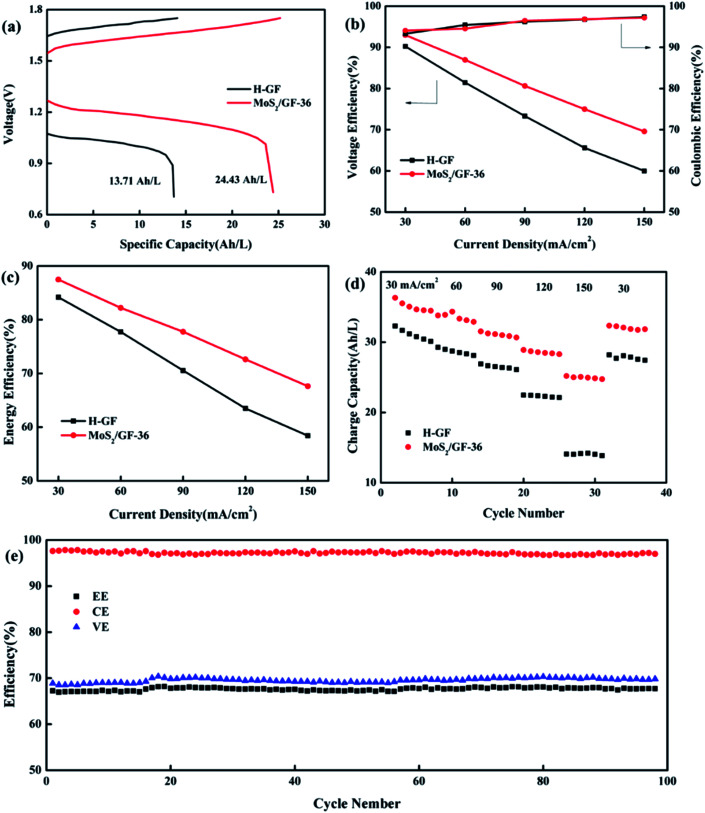
Electrochemical performance of VRFBs employing the H-GF and MoS_2_/GF-36 electrodes in single cells: (a) charge–discharge curves at 150 mA cm^−2^; (b) VE, CE, (c) EE and (d) the charge capacity at different current densities (30, 60, 90, 120, 150 mA cm^−2^). (e) EE, CE and VE of the VRFB using the MoS_2_/GF-36 electrode at 150 mA cm^−2^ for 100 charge–discharge cycles.

Based on the above results, the MoS_2_/GF-36 electrode was assembled in the battery as the positive electrode to test the charge/discharge performance, the H-GF electrode was also measured for comparison with the results shown in Fig. 7 and Table 5. Comparing the H-GF electrode, the MoS_2_/GF-36 electrode presents a lower charging voltage plateau (reducing by 100.50 mV) and a higher discharge voltage plateau (increasing by 195.10 mV), and a significant increase in the discharge capacity (an increase of 78.19%) is observed, suggesting the MoS_2_/GF-36 electrode exhibits a high electrochemical catalysis for the vanadium ions (Fig. 7(a)). For the battery efficiency, a gain in voltage efficiency (VE) and energy efficiency (EE) is found by using the MoS_2_/GF-36 electrode instead of the H-GF electrode (Fig. 7(b) and (c)). In particular, at a high current density of 150 mA cm^−2^, the MoS_2_/GF-36 electrode show a significant increase in VE and EE of 9.59% and 9.22%, respectively. However, the CE of the MoS_2_/GF-36 and H-GF electrodes are almost equal and slightly increase as the current density increases. In Fig. 7(d), since the polarization overpotential of H-GF is significantly increased, the difference in the charge capacity of the two electrodes remarkably increases as the current density increases. Therefore, at a high current density of 150 mA cm^−2^, the charge capacity of the MoS_2_/GF-36 electrode is 24.97 A h L^−1^, which is 1.77 times the charge capacity of H-GF (14.06 A h L^−1^). In Fig. 7(e), the MoS_2_/GF-36 electrode is subjected to a long cycle test at a current density of 150 mA cm^−2^. After 100 cycles, the VE, CE and EE of the MoS_2_/GF-36 electrode remain almost unchanged, the MoS_2_ were well retained on the electrode (Fig. S1†), signifying the excellent long-term stability of the electrode.

**Table 5:** Efficiencies of the VRFB cell using the MoS_2_/GF-36 and H-GF electrodes at current densities of 30, 60, 90, 120 and 150 mA cm^−2^

Current density (mA cm^−2^)	MoS_2_/GF-36			H-GF		
	EE (%)	CE (%)	VE (%)	EE (%)	CE (%)	VE (%)
30	87.47	94.03	92.97	84.18	93.3	90.22
60	82.21	94.55	86.96	77.73	95.44	81.44
90	77.76	96.44	80.64	70.54	96.22	73.31
120	72.63	96.83	75.00	63.48	96.76	65.60
150	67.693	97.2	69.58	58.41	97.38	59.99

**Fig. 8:**
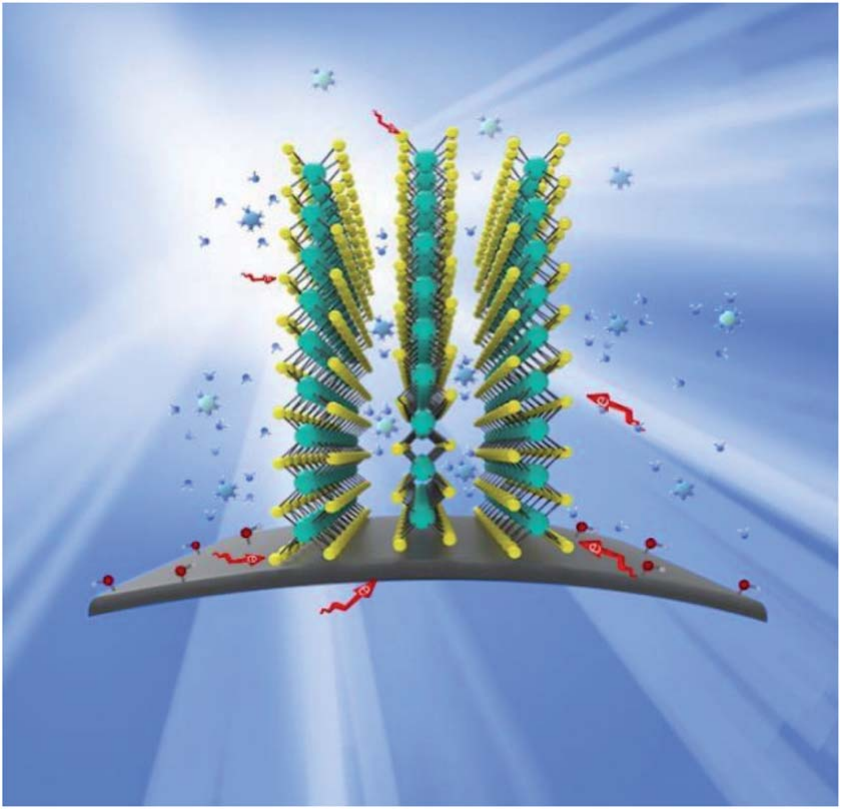
Schematic illustration of the redox reaction mechanism proposed for the VO^2+^/VO_2_^+^ redox couple on the surface of the MoS_2_/GF-36 electrode in a VRFB.

The probable catalytic mechanism on the MoS_2_/GF-36 electrode towards the VO^2+^/VO_2_^+^ redox reaction can be shown in Fig. 8. The compactly contacts through C–S bond between GF and MoS_2_, so the special structure of MoS_2_ provides effective electron transport paths for vanadium ions, allowing the electrolyte to enter the nanosheet gap. Therefore, VO^2+^/VO_2_^+^ redox reaction occurs inside and outside the nanosheet. At the same time, VO^2+^/VO_2_^+^ also promotes the redox reaction by the oxygen-containing functional group –OH binding on GF. In addition, the 3D flower structure of MoS_2_ on the surface of GF enhances the contact area between the electrode and the electrolyte, greatly shortens the diffusion distance and accelerates the charge mass transfer process. Therefore, the successful combination of GF and MoS_2_ produces extraordinary electrochemical and battery performance.

## 4. Conclusion

In summary, a safe and simple hydrothermal method was applied to grow 3D flower-like MoS_2_ nanosheets on a GF electrode for the first time. The morphology and loading of the MoS_2_ nanosheets onto the surface of the GF electrode played an important role in determining the electrochemical performance of the electrocatalyst. The electrochemical properties, reversibility, and electrical conductivity of all the MoS_2_/GF electrodes were significantly improved compared to the H-GF electrode. The kinetic studies showed that MoS_2_/GF electrodes promote the diffusion process, which promotes the VO^2+^/VO_2_^+^ redox reaction. At a high current density of 150 mA cm^−2^, the EE and the discharge capacities of the MoS_2_/GF-36 electrode were increased by 9.22% and 10.57 A h L^−1^, respectively. The charge–discharge stability test showed that the efficiency was not attenuated after 100 cycles, indicating the optimal stability of the MoS_2_-modified GF electrode in a strongly acidic electrolyte. The above results are attributed to the faster electron transfer due to the open structure of MoS_2_, the ultrawide layer spacing, and the rich oxygen-containing functional groups, which accelerate the redox reaction of the surface of the MoS_2_/GF-36 electrode. We believe this material can be a promising candidate for the development of a high performance VRFB.

## Conflicts of interest

There are no conflicts to declare.

## Supplementary Material

RA-010-D0RA02541K-s001
