# Supplementary material for: 3D flower-like molybdenum disulfide modified graphite felt as a positive material for vanadium redox flow batteries
Source: RSC Adv. 2020 May 4;10(29):17235–46. doi: 10.1039/d0ra02541k (PMC9053512; doi:10.1039/d0ra02541k)
Supplement: RA-010-D0RA02541K-s001 [file RA-010-D0RA02541K-s001.pdf]

Electronic Supplementary Material (ESI) for RSC Advances.
This journal is © The Royal Society of Chemistry 2020

3D Flower-Like Molybdenum Disulfide Modified Graphite Felt as Positive Material for Vanadium Redox Flow Battery

Lei Wang[a], Shuangyu Li[a], Dan Li[b], Qinhao Xiao[a], Wenheng Jing[a,*]

[a]State Key Laboratory of Materials-Oriented Chemical Engineering, College of Chemical Engineering, Nanjing Tech University, Nanjing 211816, China

[b]Jiangsu Jiayi Thermal Power Co., Ltd., Changzhou 213200, China

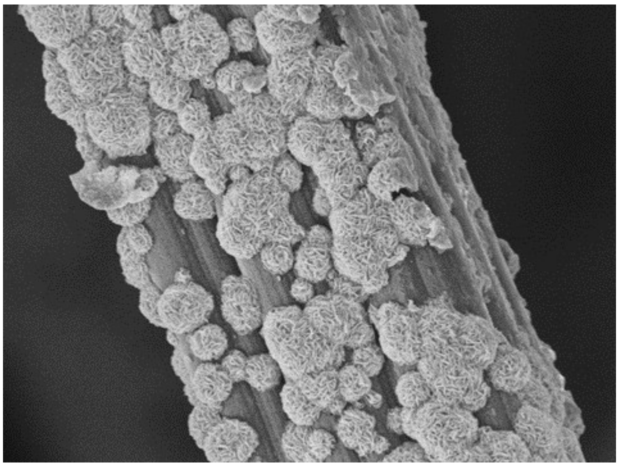

Fig.S1 The SEM images of the used MoS_2/GF electrode after long-term operation.
